# Reply to Auspurg: On the limits of “justified” model spaces

**DOI:** 10.1073/pnas.2523374122

**Published:** 2025-10-20

**Authors:** Michael Ganslmeier, Tim Vlandas

**Affiliations:** ^a^Centre for Computational Social Science, Department of Humanities, Arts and Social Sciences, University of Exeter, Exeter EX4 4PY, United Kingdom; ^b^St. Antony’s College and Department of Social Policy and Intervention, University of Oxford, Oxford OX1 2ER, United Kingdom

We are grateful to Auspurg (1) for the thoughtful engagement with our article (2). We share the author’s concern for improving robustness in the social sciences but differ on how best to achieve this. Auspurg argues that results become more reliable once the analysis is restricted to a smaller set of “justified” models. We see several difficulties with this view.

## Model Choices

What counts as justified is itself heavily contested. The widely used fixed-effects model that Auspurg advocates can amplify rather than reduce bias (3). Further, there is often no agreement on which controls should be excluded: including colliders or posttreatment variables can indeed be problematic, but omitting variables risks bias. Nor is there a consensus on country samples: for instance, should the power resource theory be tested on all OECD countries, the EU, or just Western Europe? Each choice can produce different conclusions. Theory can and should certainly inform these choices, but the empirical evidence that adjudicates between competing theories may itself be subject to the sensitivity we document. Also, our point is not that everything is unstable but that high uncertainty in the model space can lead to fragile results appearing more robust than they actually are (4).

## Measurement

There are also many reasonable disagreements over conceptualization and measurement. Developing a good quantitative indicator is hard; a perfect one that is unambiguously superior in all situations is likely impossible. Consider the longstanding literature on democracy: some researchers use Polity, others Freedom House or V-Dem. Since these indices often differ, findings about which determinants of democratization matter may end up varying widely. In most debates, multiple measures coexist, and our article shows how disagreements over measurement can produce empirical instability.

## Counterevidence

Despite excluding most combinations to focus only on the justified models, the evidence in Auspurg’s letter presented in figure 1 paradoxically confirms the lack of robustness we document in our article. When over 65% of coefficients are statistically insignificant, we cannot conclude that union density has a robust positive association with social spending. Thus, fragility dominates even within this heavily restricted justified set of models.

## Sources of Instability

Elsewhere, Auspurg and Brüderl have highlighted the importance of identifying which analytical choices drive variation (5). Our article contributes a systematic method that pinpoints the sources of instability and quantifies their impact. Our analysis of four cases using different types of datasets (i.e., country-year, regional-year, and survey data) shows that results shift far more when varying the outcome measure or the sample than when adjusting the control set. Auspurg doubts that measurement and sampling matter more than conditioning but offers no evidence in support of these doubts in our (or other) cases.

## Conclusion

In summary, Auspurg argues that we should assess robustness only after narrowing the model space to justified models. Because scholars regularly disagree on theory, measurement, and statistical assumptions, such narrowing risks assuming away what sensitivity analyses are meant to explore to begin with. Our article provides a systematic method that enables researchers to assess sensitivity openly and identify which assumptions and empirical decisions matter most for their research question.
